# The Lister Ward at Glasgow Royal Infirmary

**Published:** 1922-04

**Authors:** D. Frame

**Affiliations:** Glasgow


*To the Editor of* The Hospital and Health Review.

### The Lister Ward at Glasgow Royal Infirmary.

*To the Editor of* The Hospital and Health Review.

Sir,—There must be considerable misgiving in the minds of many people throughout the country concerning the decision come to by the managers of Glasgow Royal Infirmary, in relation to the Lister Ward. The service rendered by Lord Lister to the cause of human well-being is incalculable; but, on the other hand, the lamentable lack of sentiment shown by them is not calculated to add to the happiness of the world, or yet give a fresh impulse to those men and women who stand peculiarly in need of encouragement. I refer particularly to the medical and nursing professions. In my opinion, there is no place in the manifold associations of mankind where the spirit of sentiment is more needed than within the walls of an infirmary.

I trust, Sir, that sufficient interest will be taken in this matter, to secure the retention of the Lister Ward.

I am, &c.,

Glasgow. D. Frame.

